# 4,4-Dichloro­tricyclo­[5.4.0.0^3,5^]undeca-7,9,11-triene

**DOI:** 10.1107/S160053680801831X

**Published:** 2008-06-21

**Authors:** Wan-Li Chen, Guo-Sheng Chen, Ying-Hong Zhu, Wei-Min Mo

**Affiliations:** a Center of Analysis and Measurement, Zhejiang University of Technology, Hangzhou 310014, People’s Republic of China; b State Key Laboratory Breeding Base of Green Chemistry-Synthesis Technology, College of Chemical Engineering and Materials, Zhejiang University of Technology, Hangzhou 310014, People’s Republic of China

## Abstract

The title compound, C_11_H_10_Cl_2_, is a useful inter­mediate for the synthesis of 1*H*-cyclo­propa[*b*]naphthalene. Strain in the mol­ecule is evidenced by the fact that the cyclo­hexane ring is essentially planar and nearly coplanar with the benzene ring [dihedral angle 1.87 (18)°], and the cyclo­propyl ring is almost perpendicular to the cyclo­hexane ring [dihedral angle 70.99 (12)°]. The mol­ecules are loosely connected into one-dimensional chains by inter­molecular Cl⋯Cl inter­actions with a distance of 3.571 (1) Å. The centroid-to-centroid distance between stacked benzene rings is *ca* 5.89 Å, indicating that no π–π stacking exists in the crystal structure.

## Related literature

For related literature, see: Browne *et al.* (1974 ▶); Halton (2003 ▶).
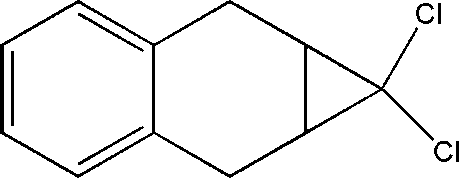


## Experimental

### 

#### Crystal data

C_11_H_10_Cl_2_
*M* *_r_* = 213.09
Monoclinic, 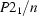

*a* = 11.598 (2) Å
*b* = 5.8920 (12) Å
*c* = 14.861 (3) Å
β = 101.97 (3)°
*V* = 993.5 (3) Å^3^
*Z* = 4
Mo *K*α radiation
μ = 0.60 mm^−1^
*T* = 298 (2) K
0.26 × 0.21 × 0.18 mm

#### Data collection

Bruker SMART 1K CCD area-detector diffractometer
Absorption correction: multi-scan (*SADABS*; Sheldrick, 2002 ▶) *T*_min_ = 0.860, *T*_max_ = 0.900
7291 measured reflections
1765 independent reflections
1363 reflections with *I* > 2σ(*I*)
*R*_int_ = 0.036

#### Refinement

*R*[*F* ^2^ > 2σ(*F* ^2^)] = 0.040
*wR*(*F* ^2^) = 0.101
*S* = 1.07
1765 reflections
118 parameters
H-atom parameters constrained
Δρ_max_ = 0.20 e Å^−3^
Δρ_min_ = −0.29 e Å^−3^

### 

Data collection: *SMART* (Bruker, 2001 ▶); cell refinement: *SAINT* (Bruker, 2001 ▶); data reduction: *SAINT*; program(s) used to solve structure: *SHELXTL* (Sheldrick, 2008 ▶); program(s) used to refine structure: *SHELXTL*; molecular graphics: *SHELXTL*; software used to prepare material for publication: *SHELXTL* and *publCIF* (Westrip, 2008 ▶).

## Supplementary Material

Crystal structure: contains datablocks global, I. DOI: 10.1107/S160053680801831X/fl2197sup1.cif

Structure factors: contains datablocks I. DOI: 10.1107/S160053680801831X/fl2197Isup2.hkl

Additional supplementary materials:  crystallographic information; 3D view; checkCIF report

## Figures and Tables

**Fig. 1.**
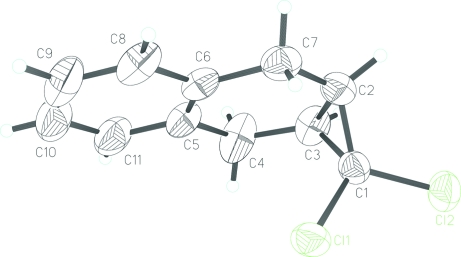
The molecular structure of (I), with displacement ellipsoids drawn at the 40% probability level. H atoms are shown as spheres of arbitrary radius.

**Fig. 2.**
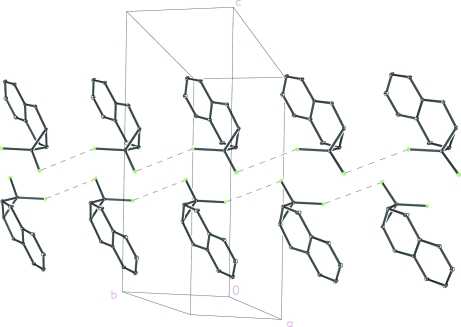
Partial packing view of (I) showing Cl···Cl interaction. H atoms have been omitted for clarity.

## supplementary crystallographic information

### Comment

The title compound, (I), is a useful intermediate for the synthesis of 1*H*-cyclopropa[*b*]naphthalene, which is a highly strained but readily accessible molecule that has been used as a building block in organic synthesis. It can undergo a variety of reactions because relief of ring strain provides a potent thermodynamic driving force for the reactions (Halton, 2003). Strain in (I) is evidenced by the fact that the cyclohexane ring is essentialy planar (Fig. 1) and nearly coplanar with the benzene ring with a dihedral angle of 1.87 (18)° and that the cyclopropyl ring is almost perpendicular to cyclohexane ring with a dihedral angle of 70.99 (12)°. The molecules pack to form one-dimensional chains connected by intermolecular Cl···Cl interactions with distance of 3.571 (1) Å (Fig. 2). The centroid to centroid distance between stacked benzene rings is *ca* 5.89 Å, which is very long indicating that no π–π stacking exists in the crystal.

### Experimental

Following the general procedure of Browne *et al.* (1974) a mixture of 1,4-dihydronaphthalene (15g, 0.115 mol), 7 ml of diethylene glycol dimethyl, and 10 ml of tetrachloroethylene was stirred magnetically and brought to 115°C, and sodium trichloroacetate (25 g, 0.144 mol) was added in 1 g portions over a period of 1 h. The mixture was stirred and maintained near boiling (115–120°C) during the addition and for an additional half hour. After separating, washing, and drying the organic phase, concentration in vacuum afforded a brown oil which was distilled to give 7,7-dichlorobenzobicyclo[4.1.0]hept-3-ene (6.85 g, 47% based on the dihydronaphthalene used) and the single crystals were obtained by evaporation of a petroleum ether solution. b.p. 389–391 K at 1 mm, m.p. 328–330 K. ^1^H NMR (400 MHz, CDCl_3_): δ 2.03–2.05 (m, 2H); 2.76–2.82 (m, 2H), 3.18–3.25 (m, 2H), 7.10 (s, 4H).

### Refinement

H atoms were positioned geometrically and treated as riding, with C—H bond lengths constrained to 0.93 (aromatic CH), 0.97 Å (methylene CH2) and with *U*_iso_(H) = 1.2Ueq (carrier aromatic C and methylene C).

### Crystal data

**Table:**

C_11_H_10_Cl_2_	*F*_000_ = 440
*M**_r_* = 213.09	*D*_x_ = 1.425 Mg m^−^^3^
Monoclinic, *P*2_1_/*n*	Mo *K*α radiation λ = 0.71073 Å
Hall symbol: -P 2yn	Cell parameters from 6708 reflections
*a* = 11.598 (2) Å	θ = 3.0–27.5º
*b* = 5.8920 (12) Å	µ = 0.60 mm^−^^1^
*c* = 14.861 (3) Å	*T* = 298 (2) K
β = 101.97 (3)º	Chunk, colourless
*V* = 993.5 (3) Å^3^	0.26 × 0.21 × 0.18 mm
*Z* = 4	

### Data collection

**Table:**

Bruker SMART 1K CCD area-detector diffractometer	1765 independent reflections
Radiation source: fine-focus sealed tube	1363 reflections with *I* > 2σ(*I*)
Monochromator: graphite	*R*_int_ = 0.036
*T* = 298(2) K	θ_max_ = 25.1º
φ and ω scans	θ_min_ = 3.6º
Absorption correction: multi-scan(SADABS; Sheldrick, 2002)	*h* = −13→13
*T*_min_ = 0.860, *T*_max_ = 0.900	*k* = −7→6
7291 measured reflections	*l* = −17→17

### Refinement

**Table:**

Refinement on *F*^2^	Secondary atom site location: difference Fourier map
Least-squares matrix: full	Hydrogen site location: inferred from neighbouring sites
*R*[*F*^2^ > 2σ(*F*^2^)] = 0.040	H-atom parameters constrained
*wR*(*F*^2^) = 0.101	*w* = 1/[σ^2^(*F*_o_^2^) + (0.0595*P*)^2^ + 0.3188*P*] where *P* = (*F*_o_^2^ + 2*F*_c_^2^)/3
*S* = 1.08	(Δ/σ)_max_ = 0.001
1765 reflections	Δρ_max_ = 0.20 e Å^−^^3^
118 parameters	Δρ_min_ = −0.29 e Å^−^^3^
Primary atom site location: structure-invariant direct methods	Extinction correction: none

### Special details

**Table:**

Geometry. All e.s.d.'s (except the e.s.d. in the dihedral angle between two l.s. planes) are estimated using the full covariance matrix. The cell e.s.d.'s are taken into account individually in the estimation of e.s.d.'s in distances, angles and torsion angles; correlations between e.s.d.'s in cell parameters are only used when they are defined by crystal symmetry. An approximate (isotropic) treatment of cell e.s.d.'s is used for estimating e.s.d.'s involving l.s. planes.
Refinement. Refinement of *F*^2^ against ALL reflections. The weighted *R*-factor *wR* and goodness of fit *S* are based on *F*^2^, conventional *R*-factors *R* are based on *F*, with *F* set to zero for negative *F*^2^. The threshold expression of *F*^2^ > σ(*F*^2^) is used only for calculating *R*-factors(gt) *etc*. and is not relevant to the choice of reflections for refinement. *R*-factors based on *F*^2^ are statistically about twice as large as those based on *F*, and *R*- factors based on ALL data will be even larger.

### Fractional atomic coordinates and isotropic or equivalent isotropic displacement parameters (Å^2^)

**Table:**

	*x*	*y*	*z*	*U*_iso_*/*U*_eq_	
Cl1	0.21220 (6)	1.03963 (10)	0.93462 (4)	0.0652 (2)	
Cl2	0.23062 (6)	0.60334 (11)	1.02480 (4)	0.0674 (2)	
C1	0.1572 (2)	0.7642 (4)	0.92994 (14)	0.0491 (5)	
C2	0.1202 (2)	0.6470 (4)	0.84012 (15)	0.0554 (6)	
H2	0.1358	0.4834	0.8414	0.066*	
C3	0.0301 (2)	0.7219 (5)	0.89436 (15)	0.0614 (6)	
H3	−0.0032	0.5987	0.9253	0.074*	
C4	−0.0539 (2)	0.9095 (6)	0.86004 (18)	0.0837 (10)	
H4A	−0.0484	1.0214	0.9086	0.100*	
H4B	−0.1332	0.8481	0.8486	0.100*	
C5	−0.0371 (2)	1.0298 (4)	0.77408 (14)	0.0574 (6)	
C6	0.0445 (2)	0.9597 (4)	0.72401 (14)	0.0515 (6)	
C7	0.1263 (2)	0.7628 (4)	0.75120 (15)	0.0596 (6)	
H7A	0.2064	0.8157	0.7551	0.072*	
H7B	0.1101	0.6507	0.7024	0.072*	
C8	0.0505 (2)	1.0778 (5)	0.64359 (16)	0.0687 (7)	
H8	0.1043	1.0319	0.6088	0.082*	
C9	−0.0210 (3)	1.2590 (6)	0.61490 (19)	0.0821 (9)	
H9	−0.0157	1.3343	0.5609	0.099*	
C10	−0.1007 (3)	1.3307 (6)	0.6654 (2)	0.0828 (9)	
H10	−0.1490	1.4551	0.6464	0.099*	
C11	−0.1080 (2)	1.2166 (5)	0.74404 (17)	0.0727 (8)	
H11	−0.1619	1.2651	0.7784	0.087*	

### Atomic displacement parameters (Å^2^)

**Table:**

	*U*^11^	*U*^22^	*U*^33^	*U*^12^	*U*^13^	*U*^23^
Cl1	0.0862 (5)	0.0485 (4)	0.0576 (4)	−0.0129 (3)	0.0072 (3)	−0.0011 (3)
Cl2	0.0776 (5)	0.0628 (4)	0.0593 (4)	0.0120 (3)	0.0079 (3)	0.0130 (3)
C1	0.0556 (14)	0.0420 (12)	0.0501 (12)	0.0008 (10)	0.0114 (10)	0.0030 (10)
C2	0.0623 (15)	0.0446 (12)	0.0582 (13)	−0.0039 (11)	0.0103 (11)	−0.0032 (11)
C3	0.0523 (15)	0.0740 (17)	0.0590 (13)	−0.0081 (12)	0.0144 (11)	0.0151 (13)
C4	0.0592 (17)	0.133 (3)	0.0629 (15)	0.0237 (17)	0.0224 (13)	0.0273 (17)
C5	0.0473 (14)	0.0767 (17)	0.0449 (12)	0.0016 (12)	0.0018 (10)	0.0006 (12)
C6	0.0462 (13)	0.0645 (15)	0.0408 (11)	−0.0113 (11)	0.0024 (9)	−0.0034 (11)
C7	0.0610 (16)	0.0676 (16)	0.0516 (13)	0.0007 (12)	0.0146 (11)	−0.0096 (12)
C8	0.0583 (16)	0.099 (2)	0.0470 (13)	−0.0164 (15)	0.0058 (11)	0.0071 (14)
C9	0.073 (2)	0.101 (2)	0.0627 (16)	−0.0193 (18)	−0.0080 (15)	0.0304 (16)
C10	0.071 (2)	0.085 (2)	0.0794 (19)	0.0047 (16)	−0.0157 (16)	0.0134 (17)
C11	0.0582 (16)	0.093 (2)	0.0596 (15)	0.0126 (15)	−0.0040 (12)	−0.0030 (15)

### Geometric parameters (Å, °)

**Table:**

Cl1—C1	1.740 (2)	C5—C11	1.391 (4)
Cl2—C1	1.764 (2)	C6—C8	1.397 (3)
C1—C3	1.481 (3)	C6—C7	1.500 (3)
C1—C2	1.485 (3)	C7—H7A	0.9700
C2—C7	1.502 (3)	C7—H7B	0.9700
C2—C3	1.512 (3)	C8—C9	1.365 (4)
C2—H2	0.9800	C8—H8	0.9300
C3—C4	1.491 (4)	C9—C10	1.372 (4)
C3—H3	0.9800	C9—H9	0.9300
C4—C5	1.508 (3)	C10—C11	1.366 (4)
C4—H4A	0.9700	C10—H10	0.9300
C4—H4B	0.9700	C11—H11	0.9300
C5—C6	1.383 (3)		
			
C3—C1—C2	61.31 (16)	C6—C5—C11	119.3 (2)
C3—C1—Cl1	120.06 (18)	C6—C5—C4	122.6 (2)
C2—C1—Cl1	120.33 (15)	C11—C5—C4	118.2 (2)
C3—C1—Cl2	118.33 (16)	C5—C6—C8	118.2 (2)
C2—C1—Cl2	118.03 (16)	C5—C6—C7	123.4 (2)
Cl1—C1—Cl2	110.92 (12)	C8—C6—C7	118.4 (2)
C1—C2—C7	121.5 (2)	C6—C7—C2	116.50 (19)
C1—C2—C3	59.20 (15)	C6—C7—H7A	108.2
C7—C2—C3	120.0 (2)	C2—C7—H7A	108.2
C1—C2—H2	115.0	C6—C7—H7B	108.2
C7—C2—H2	115.0	C2—C7—H7B	108.2
C3—C2—H2	115.0	H7A—C7—H7B	107.3
C1—C3—C4	121.9 (2)	C9—C8—C6	121.5 (3)
C1—C3—C2	59.49 (15)	C9—C8—H8	119.3
C4—C3—C2	120.6 (2)	C6—C8—H8	119.3
C1—C3—H3	114.6	C8—C9—C10	120.3 (3)
C4—C3—H3	114.6	C8—C9—H9	119.9
C2—C3—H3	114.6	C10—C9—H9	119.9
C3—C4—C5	116.6 (2)	C11—C10—C9	119.0 (3)
C3—C4—H4A	108.1	C11—C10—H10	120.5
C5—C4—H4A	108.1	C9—C10—H10	120.5
C3—C4—H4B	108.1	C10—C11—C5	121.8 (3)
C5—C4—H4B	108.1	C10—C11—H11	119.1
H4A—C4—H4B	107.3	C5—C11—H11	119.1
			
C3—C1—C2—C7	108.5 (3)	C3—C4—C5—C11	−175.2 (2)
Cl1—C1—C2—C7	−1.4 (3)	C11—C5—C6—C8	−1.3 (3)
Cl2—C1—C2—C7	−142.7 (2)	C4—C5—C6—C8	177.8 (2)
Cl1—C1—C2—C3	−110.0 (2)	C11—C5—C6—C7	179.1 (2)
Cl2—C1—C2—C3	108.8 (2)	C4—C5—C6—C7	−1.8 (4)
C2—C1—C3—C4	−109.2 (2)	C5—C6—C7—C2	−2.9 (3)
Cl1—C1—C3—C4	1.2 (3)	C8—C6—C7—C2	177.6 (2)
Cl2—C1—C3—C4	142.6 (2)	C1—C2—C7—C6	−66.7 (3)
Cl1—C1—C3—C2	110.39 (19)	C3—C2—C7—C6	3.4 (3)
Cl2—C1—C3—C2	−108.28 (19)	C5—C6—C8—C9	0.6 (4)
C7—C2—C3—C1	−111.0 (2)	C7—C6—C8—C9	−179.8 (2)
C1—C2—C3—C4	111.4 (3)	C6—C8—C9—C10	0.4 (4)
C7—C2—C3—C4	0.4 (4)	C8—C9—C10—C11	−0.7 (4)
C1—C3—C4—C5	66.1 (3)	C9—C10—C11—C5	−0.1 (4)
C2—C3—C4—C5	−4.8 (4)	C6—C5—C11—C10	1.1 (4)
C3—C4—C5—C6	5.6 (4)	C4—C5—C11—C10	−178.1 (3)
